# Multiple sclerosis relapse following gamma knife radiosurgery for meningioma in a patient treated with natalizumab: case report

**DOI:** 10.3389/fonc.2026.1959731

**Published:** 2026-10-09

**Authors:** Maryna Turbinska, Nabeela Nathoo, Penelope Smyth

**Affiliations:** Division of Neurology, University of Alberta, Edmonton, AB, Canada

**Keywords:** gamma knife radiosurgery, meningioma, multiple sclerosis, natalizumab, radiation neurotoxicity

## Abstract

**Objectives:**

We describe a case of a patient with multiple sclerosis (MS) stable on natalizumab who developed widespread gadolinium-enhancing lesions and clinical relapse seven and eight months after gamma knife radiosurgery (GKRS) for left cerebellar convexity meningioma. We explore how increased blood–brain barrier (BBB) permeability post-GKRS could potentially interfere with the lymphocyte anti-trafficking effect of natalizumab and result in new central nervous system (CNS) inflammation distant from the radiation site.

**Methods:**

Comprehensive analysis of the patient’s chart, brain and spine imaging, serum and cerebrospinal fluid (CSF) profiles was conducted. We compiled a graphical representation of treatment changes, radiologic findings, and GKRS initiation within one timeframe. We conducted a structured literature review to support the discussion of this case and hypothesize possible pathophysiological mechanisms contributing to clinical and radiological findings.

**Results:**

Seven months after GKRS, follow-up brain magnetic resonance imaging (MRI) demonstrated multiple gadolinium-enhancing lesions in both cerebral hemispheres, and repeat imaging one month later revealed new gadolinium-enhancing lesions, correlating with the patient’s clinical relapse. Alternative causes of disease progression, including radiation-induced demyelination, radionecrosis, opportunistic infections, progressive multifocal leukoencephalopathy (PML), and development of anti-natalizumab antibodies, were explored and considered less likely. The patient was switched to ofatumumab with subsequent clinical and radiological stability.

**Discussion:**

Given extensive new inflammatory lesions far from the GKRS site, it seems unlikely that the sole mechanism was radiation-induced neurotoxicity. We hypothesize that late delayed radiation side effects caused by GKRS may have enabled peripheral lymphocytes to enter the CNS, counteracting the effects of natalizumab on blocking lymphocyte trafficking through the BBB and possibly contributing to MS relapse.

## Introduction

Gamma knife radiosurgery (GKRS) is a minimally invasive alternative to resection of meningiomas in surgically challenging or high-risk locations (1, 2). Neurotoxic effects of cranial radiosurgery are well known and often described months to years after exposure (3). Patients with multiple sclerosis (MS) may experience even higher rates of radiation-induced neurotoxicity compared to those without demyelinating disease (4, 5). We present a case of a patient with MS treated with the lymphocyte anti-trafficking agent natalizumab, who developed widespread gadolinium-enhancing lesions and clinical relapse several months after single-fraction GKRS for meningioma. We explore the potential relationship between late delayed radiation neurotoxicity and new demyelination as a unique aspect of this case, with the main hypothesis being that lymphocytes were able to traffic into the central nervous system (CNS) after blood-brain barrier (BBB) disruption from GKRS, despite natalizumab’s anti-trafficking effects.

## Methods

Data were extracted from the patient’s chart, including disease timecourse, treatment outcomes, clinical findings, brain and spinal magnetic resonance imaging (MRI), serum and cerebrospinal fluid (CSF) profiles. We created a graphic representation of treatment changes and radiologic stability to analyze treatment response and pathophysiological mechanisms that may have triggered the development of new inflammatory demyelinating lesions. Additionally, we conducted a structured literature review to support the discussion of this case, including searches across PubMed, Scopus, and Google Scholar databases for articles published up to February 2026, using the following terms: “meningioma treatment guidelines”, “radiosurgery” and “blood-brain barrier disruption”, “multiple sclerosis” and “gamma knife radiosurgery”, “natalizumab” and “blood-brain barrier disruption”, “natalizumab” and “radiosurgery”, “radiosurgery” and “demyelination”, “radiation-induced neurotoxicity stages”, “radiotherapy fractionation” and “radionecrosis”.

### Standard protocol approvals, registrations, and patient consents

This case report did not require ethical board approval. Written informed consent was obtained from the patient for participation and publication of this case report.

### Data availability statement

All data needed to evaluate the conclusions in the paper are presented in the main manuscript. Additional data related to this paper may be requested from the corresponding author upon reasonable request.

## Results

A 49-year-old female was diagnosed with relapsing-remitting MS in 2008, initially switching multiple disease-modifying therapies due to side effects and breakthrough inflammation (**Figure 1**). The patient achieved clinical and radiological stability while she was treated with natalizumab until she underwent GKRS in February 2023. She was treated with the standard interval dosing (every 4 weeks) of natalizumab 300 mg intravenously in 2014-2017. In 2017, she moved out of province and was started on fingolimod, but was found to have 3 gadolinium-enhancing lesions and a clinical relapse in 2019, so she was switched back to natalizumab standard interval dosing. Due to prolonged clinical and radiological stability, the patient was started on extended interval dosing (every 6 weeks) in 2021 until September 2023 without interruption, missed doses, or adverse reactions. Pertinent medical history was significant for a left cerebellar convexity meningioma diagnosed in 2020. MRI of the brain in October 2022 showed progression of meningioma with no new demyelinating lesions. Multiple bilateral infratentorial, juxtacortical, periventricular, and subcortical lesions were present at baseline; cervical and thoracic spine MRIs were not done at that time. Her baseline Expanded Disability Status Scale (EDSS) was 3.5 with balance issues, bowel and bladder urgency. In February 2023, the patient was assessed by neurosurgery and elected to proceed with GKRS. At that time, MRI of the brain showed a 1.5 cm meningioma along the left cerebellar convexity with invasion into the left transverse/sigmoid sinus junction, which had slightly increased in size compared to the previous brain MRI. She received natalizumab 25 days before and 22 days after GKRS. GKRS treatment parameters were: treated volume of 1.651 cm³, margin dose of 14 Gy, prescription isodose of 48%, maximum dose of 29.2 Gy, 25 isocenters in a single fraction. Total beam-on time was 26.3 minutes. The patient received 4 mg of dexamethasone intravenously once before the procedure. She tolerated the procedure well, requiring one break in beam-on time. Follow-up MRI of the brain in September 2023 showed multiple gadolinium-enhancing ovoid lesions (**Figure 2**): four juxtacortical, subcortical, and periventricular lesions in the left frontal lobe, two juxtacortical and subcortical lesions in the left parietal lobe, and one right periventricular lesion. Clinically, the patient noted mild cognitive decline and fatigue with no focal neurological deficits. She declined a course of pulse corticosteroid therapy due to concerns about weight gain and was switched to standard interval dosing of natalizumab, which she continued until March 2024. Repeat brain, cervical spine, and thoracic spine MRI one month later (October 2023) revealed two new large lesions in the right frontal subcortical white matter, demonstrating ring gadolinium enhancement (**Figure 3**) with multiple non-enhancing cervical spine lesions. Notably, all new lesions developed outside of the radiation field. At that time, the patient developed a clinical relapse with left foot weakness that improved in two weeks. Electromyography (EMG)/nerve conduction studies (NCS) showed reduced activation despite normal recruitment of the left tibialis anterior, suggesting an underlying central lesion impairing activation. Serum anti-natalizumab antibodies were negative in September 2023. John Cunningham (JC) virus antibodies were negative before GKRS in September 2022 and after the procedure in September 2023. Serologic testing was negative for hepatitis A, hepatitis B, hepatitis C, human immunodeficiency virus (HIV), and Varicella-Zoster Virus (VZV) in October 2023. CSF analysis in November 2023 revealed a normal white blood cell (WBC) count, positive oligoclonal bands (OCB), negative polymerase chain reaction (PCR) for JC virus, negative fungal stain and culture, and negative Gram stain and culture. Mycobacterial culture and AFB stain, Cryptococcal antigen, Herpes Simplex Virus (HSV), Varicella-Zoster Virus (VZV), Enterovirus, and Parechovirus testing were negative as well. In March 2024, four weeks after her last natalizumab dose, she was switched to ofatumumab (anti-CD20 monoclonal antibody) 20 mg subcutaneous injections once weekly for the first 3 weeks of treatment, followed by once-monthly injections. She has continued this regimen since then and has had no clinical or radiological relapses. She had a repeat MRI of the brain, cervical spine, and thoracic spine in September 2024 with no changes compared to previous imaging. There has been no change in the size of the meningioma and no new gadolinium-enhancing lesions on brain MRI at the last follow-up in September 2025.

**Figure 1:**
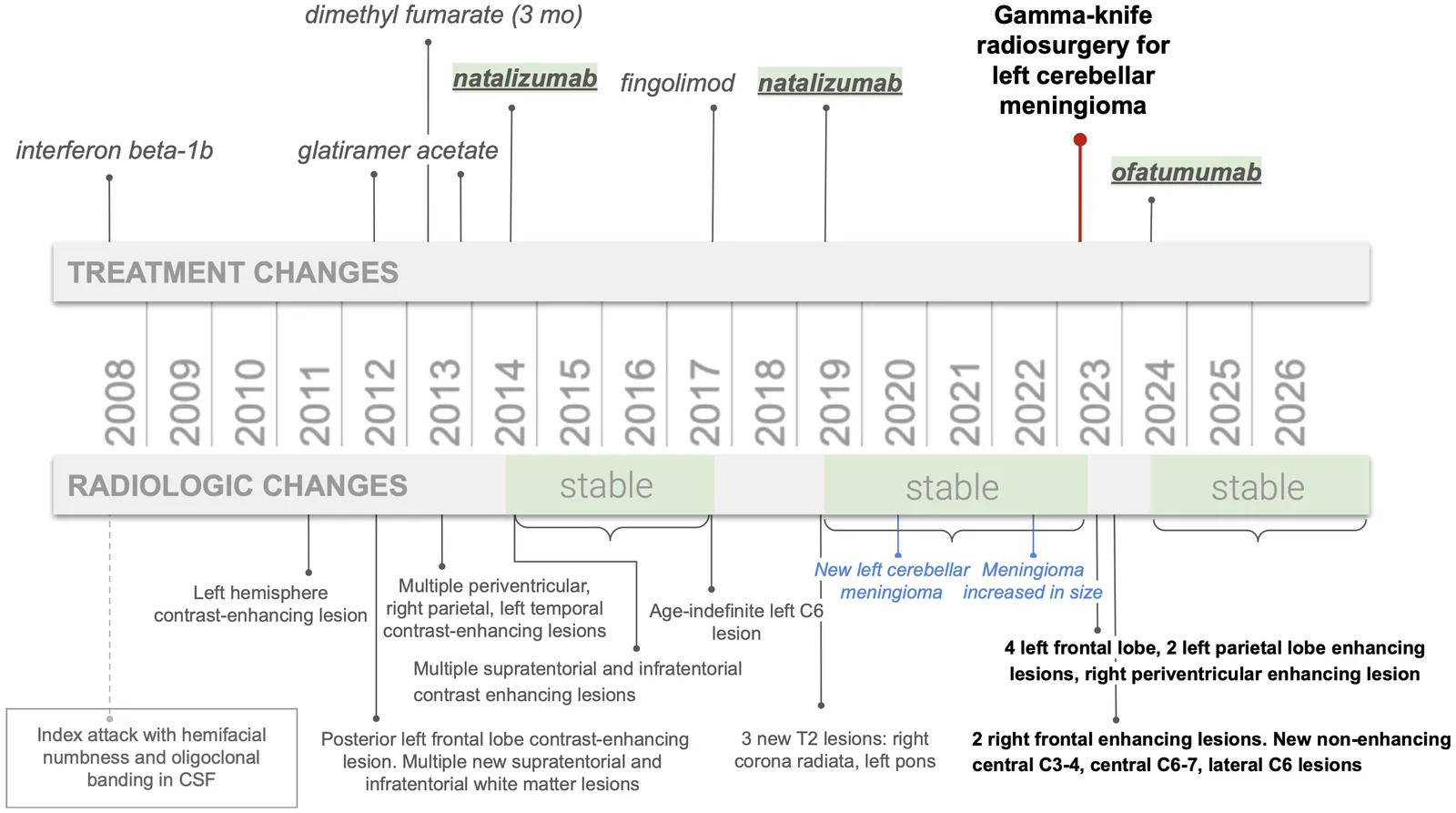
Timeline of DMT initiation for RRMS, GKRS for meningioma, and radiological findings. DMT, disease-modifying therapy; RRMS, relapsing-remitting multiple sclerosis; GKRS, gamma knife radiosurgery.

**Figure 2:**
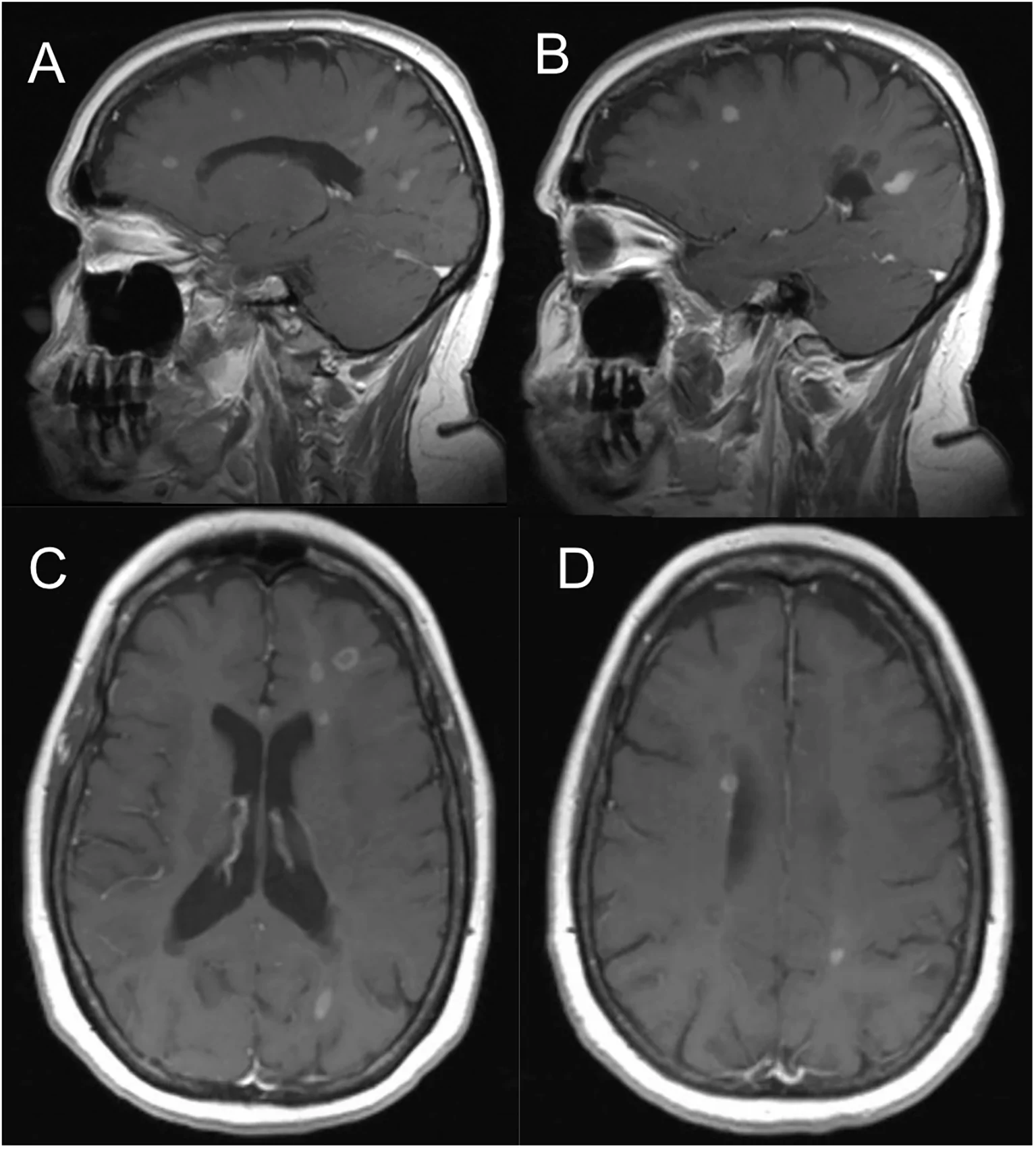
MRI brain 7 months after GKRS: multiple juxtacortical, subcortical and periventricular contrast-enhancing lesions in the left frontal lobe, juxtacortical and subcortical contrast-enhancing lesions in the left parietal lobe, right periventricular contrast-enhancing lesion on sagittal **(A, B)** and axial **(C, D)** post-contrast T1-weighted MRI. MRI, magnetic resonance imaging; GKRS, gamma knife radiosurgery.

**Figure 3:**
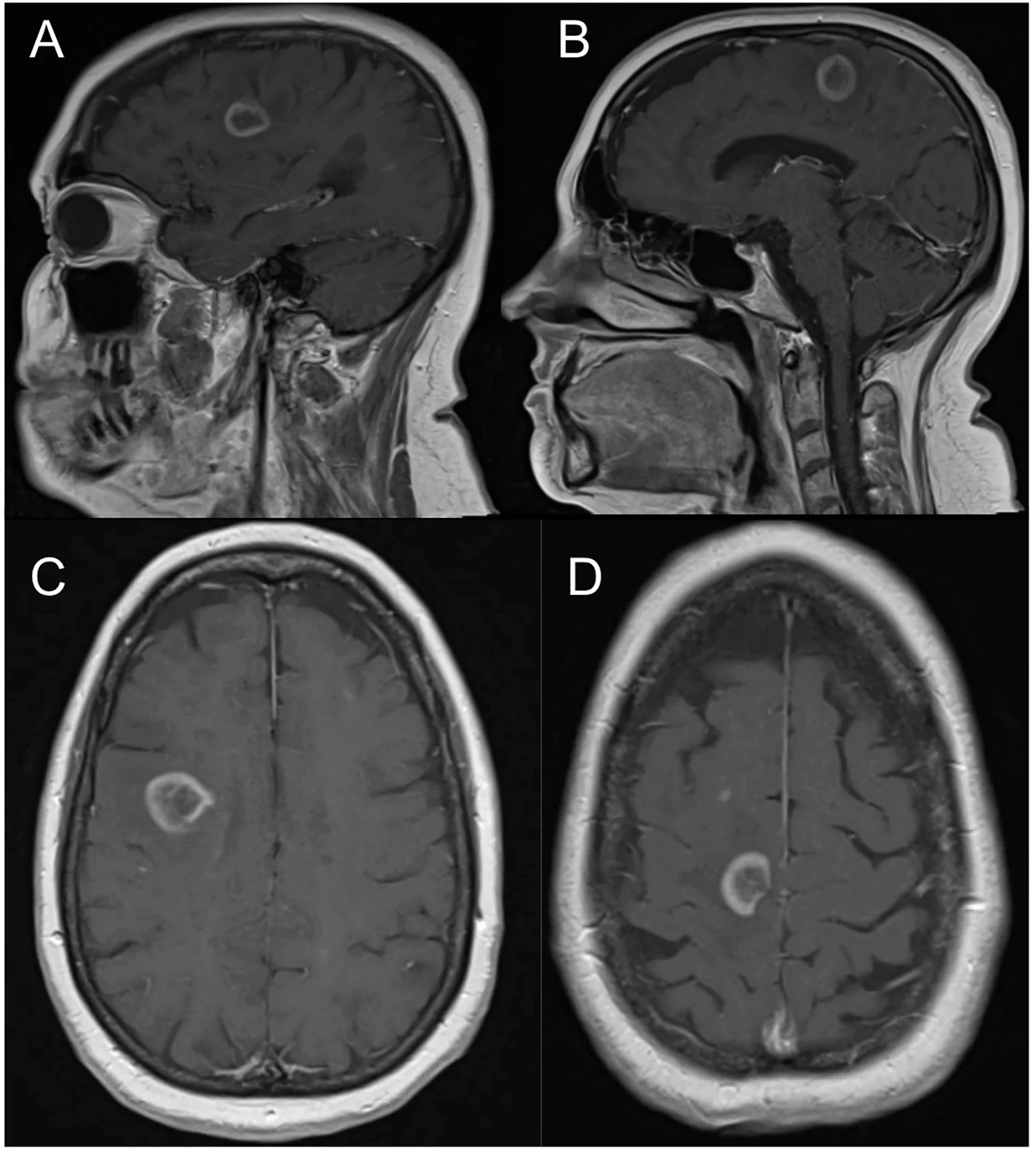
MRI brain 8 months after GKRS: Two large right frontal subcortical contrast-enhancing lesions on sagittal **(A, B)** and axial **(C, D)** post-contrast T1-weighted MRI. MRI, magnetic resonance imaging; GKRS, gamma knife radiosurgery.

## Discussion

Radiation neurotoxicity is classified as acute (hours to days), early delayed (within 6 months), and late delayed (more than 6 months) (6, 7). Acute endothelial damage to the BBB, especially with higher doses in a single fraction (8), or compression of adjacent venous structures (9) could lead to acute radiation neurotoxicity with vasogenic edema seen on MRI (10), in some studies but not others, and without appreciable trends across studies (11). Our patient was found to have bilateral gadolinium-enhancing lesions 7 and 8 months after GKRS, raising the possibility of early delayed or late delayed radiation neurotoxicity in the differential diagnosis. In early delayed radiation-induced neurotoxicity, glial cells are affected, and oligoprogenitor cells undergo apoptosis, leading to radiation-induced demyelination that is usually transient. At this point, MRI of the brain usually reveals transient nonenhancing white matter T2 hyperintensities or new contrast-enhancing lesions in close proximity to the irradiated site (10). In our case, all the new lesions developed bilaterally outside the radiation field, making radiation-induced demyelination less likely. Late delayed radiation neurotoxicity usually presents with leukoencephalopathy or radiation necrosis due to chronic vasogenic edema with hypoxia, vascular endothelial growth factor (VEGF) upregulation, post-radiation vasculopathy, and white matter inflammation or necrosis (6). Radionecrosis typically develops within the white matter adjacent to the irradiated site and nowadays is less common due to improved safety of irradiation protocols (6). MRI usually shows enhancing mass lesions with central necrosis and edema within the radiation field, often with a “spreading wavefront” appearance and central nonenhancing necrotic components (10), which were not observed in our patient. PML is also included in our differential diagnosis as the patient was treated with natalizumab for more than 24 months. MRI findings usually demonstrate diffuse, symmetric, and confluent T2 white matter hyperintensities with irregular shape and ill-defined borders, multiple punctate areas of T2-weighted hyperintensities (“Milky Way sign”) (12), or cerebellar white matter T2-weighted hyperintense lesions (“shrimp sign”) that typically do not enhance or cause mass effect (13). Most importantly, negative PCR for JC virus made PML diagnosis unlikely in our case. Additionally, negative CSF infectious studies (bacterial, viral, and opportunistic PCRs), and absence of systemic clinical features made CNS infection unlikely in this case. Absence of surrounding vasogenic edema on MRI also allowed us to suggest that demyelination is more likely to explain the current patient presentation rather than abscess or neoplasm (14). Multifocal gadolinium-enhancing ovoid lesions arising outside the radiation field across ≥3 MS-typical locations (juxtacortical, subcortical, and periventricular) with positive OCBs, and simultaneous clinical relapse in our case are highly suspicious for actively relapsing MS. The patient was compliant with her natalizumab regimen, and her anti-natalizumab antibodies were negative, making impaired treatment efficacy less likely.

Mechanistically, it is known that in late delayed radiation neurotoxicity, VEGF promotes the expression of intercellular adhesion molecule 1 (ICAM-1) (15, 16), a dominant molecule that drives leukocyte adhesion and further transmigration through activated endothelium (17). In a patient with a pre-existing demyelinating disorder, activated myelin-reactive T lymphocytes that have crossed the BBB may be reactivated by antigen-presenting cells, cytokines, and chemokines produced by immune and glial cells, leading to amplification of the inflammatory cascade within the CNS (18), which could possibly lead to bilateral demyelination in the brain. Moreover, ICAM-1 itself could potentially mediate the release of proinflammatory cytokines in glia, contributing to the toxic microenvironment of the irradiated CNS (19). Also, a recent study from Mayo Clinic in which 21% of patients with MS had new, mostly asymptomatic demyelinating lesions post-radiation found that most of the lesions were outside of the radiation volumes that were thought to be likely related to the radiation proinflammatory effects, with increased systemic release of TNF-α and Il-1β (20). This altogether has the potential to explain the bilateral appearance of the gadolinium-enhancing lesions far from the irradiated site.

The patient had no MS relapses for 4 years while treated with the lymphocyte anti-trafficking agent natalizumab before undergoing GKRS. Natalizumab prevents lymphocytes from entering the BBB, but it is unknown whether radiation-induced BBB disruption impacts its efficacy. Some studies show that BBB permeability may predict suboptimal treatment response, particularly with natalizumab (21). Natalizumab is supposed to inhibit lymphocyte entry into the CNS mostly by binding to its α4 chain of α4β1 integrin, preventing it from adhering to endothelial vascular cell adhesion molecule 1 (VCAM-1) (22), but there is no data demonstrating the direct impact of natalizumab on further leukocyte adhesion mediated by endothelial ICAM-1. We are hypothesizing that the molecular pathology of chronic radiation brain injury could potentially be misaligned with the mechanism of action of natalizumab and impact its therapeutic effect (**Figure 4**), emphasizing the need for further larger studies. There is an observation (23) showing that high levels of endothelial ICAM-1 impair the lymphocyte anti-trafficking effects of natalizumab, allowing pathogenic T cells to enter the CNS despite natalizumab blockade of very late antigen-4 (VLA-4), but the applicability of this study to the present case is limited, as these effects were only demonstrated *in vitro*.

**Figure 4:**
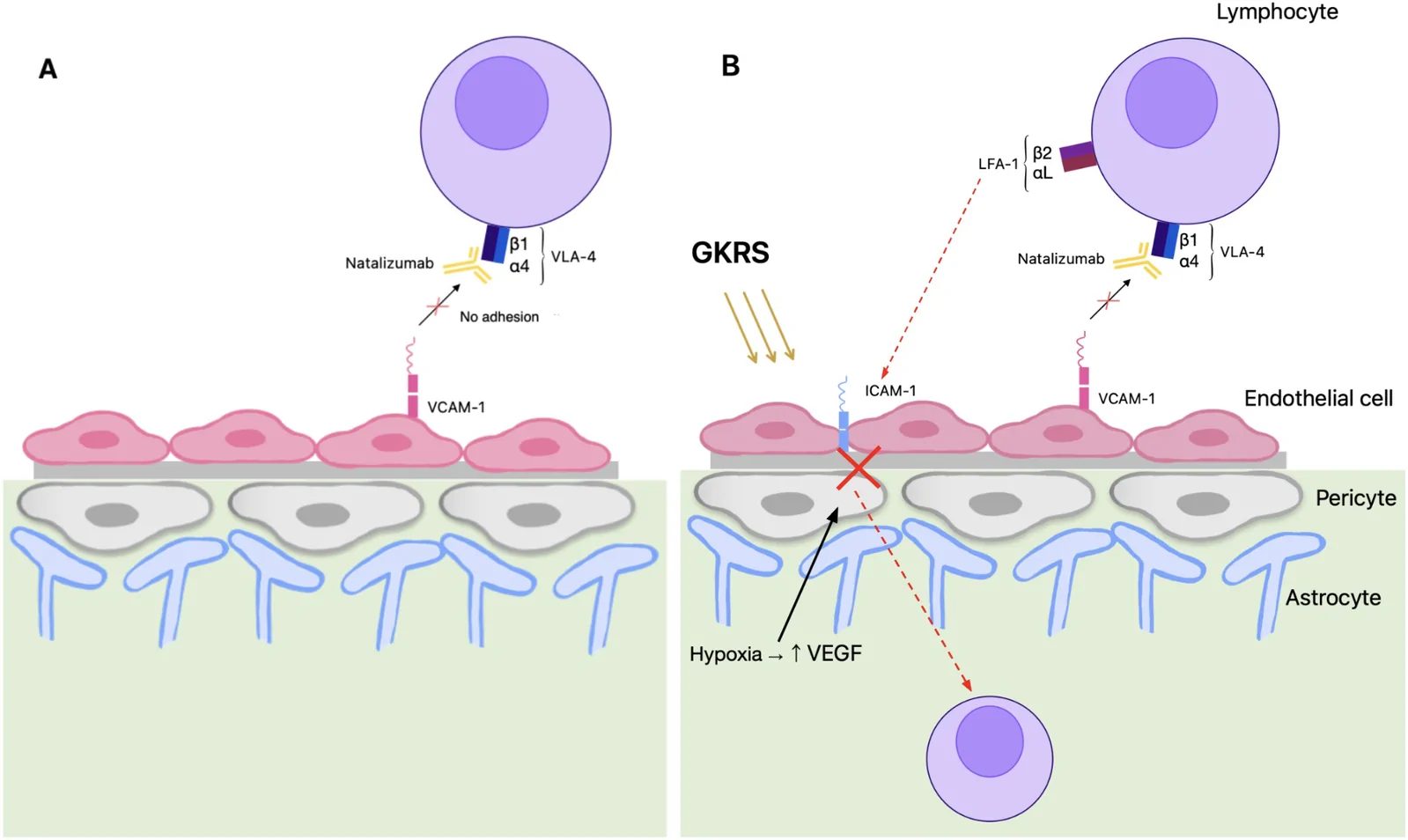
Hypothesized mechanism of neuroinflammation in the present setting: **(A)** Natalizumab blockage of α4 integrin prevents lymphocyte binding to VCAM-1 and trafficking through the BBB. **(B)** Hypoxia-induced ICAM-1 expression triggered by VEGF in late delayed radiation neurotoxicity could potentially lead to lymphocyte adhesion despite α4 integrin blockage by natalizumab. GKRS, gamma-knife radiosurgery; BBB, blood-brain barrier; VLA-4: very late antigen-4, LFA-1: lymphocyte function-associated antigen-1, VEGF, vascular endothelial growth factor; ICAM-1, intercellular adhesion molecule 1; VCAM-1, vascular cell adhesion molecule 1.

We hypothesize that the combination of natalizumab use along with the effects of late radiation neurotoxicity impacted BBB permeability following single-fraction GKRS, potentially allowing the lymphocytes to enter the CNS, causing the development of the widespread gadolinium-enhancing lesions and MS relapse. This case report raises the possibility that persons with MS on natalizumab who are considering pursuing GKRS may benefit from close individualized clinical and radiological monitoring after GKRS. It has been suggested that the use of a high-efficacy disease-modifying therapy (DMT) following radiation may provide an additional layer of protection against the development of new demyelinating lesions (20). In our case, the patient achieved prolonged clinical and radiological stability when started on an anti-CD20 monoclonal antibody that acts peripherally, rather than centrally, and the efficacy of which is unlikely to depend on BBB permeability (24). However, given the single-case nature of this report and the absence of an established causal relationship, no definitive recommendations are suggested based on these clinical observations alone.

### Limitations

Given the limited scope of the case report, generalizability is limited, and larger studies are needed to determine the validity of our hypotheses. MRI brain from 2017 to 2019 was not available for review, as the patient moved out of the province; MRI reports were used instead. MRI brain was not performed until eight months after GKRS, which affects the accurate assessment of the onset of new lesions in terms of true timelines. In the absence of direct evaluation of BBB permeability and natalizumab pharmacodynamics, the observed association cannot be confirmed to be causal.

## Patient perspective

The patient shared her perspective and emphasized the importance of publishing this clinical note to highlight potential risks of GKRS in patients with MS who are on natalizumab.

## Data Availability

The original contributions presented in the study are included in the article/**Supplementary Material**. Further inquiries can be directed to the corresponding author.

## Supplementary material

The Supplementary Material for this article can be found onlineat: https://www.frontiersin.org/articles/10.3389/fonc.2026.1959731/full#supplementary-material
